# Large scale validation of an early-age eye-tracking biomarker of an autism spectrum disorder subtype

**DOI:** 10.1038/s41598-022-08102-6

**Published:** 2022-03-11

**Authors:** Teresa H. Wen, Amanda Cheng, Charlene Andreason, Javad Zahiri, Yaqiong Xiao, Ronghui Xu, Bokan Bao, Eric Courchesne, Cynthia Carter Barnes, Steven J. Arias, Karen Pierce

**Affiliations:** 1 Autism Center of Excellence, Department of Neurosciences, University of California, San Diego, 8110 La Jolla Shores Dr., La Jolla, CA 92037 USA; 2 Department of Bioinformatics and Systems Biology, University of California, San Diego, La Jolla, CA USA; 3 Herbert Wertheim School of Public Health and Department of Mathematics, University of California, San Diego, La Jolla, CA USA

**Keywords:** Autism spectrum disorders, Diagnostic markers, Paediatric research

## Abstract

Few clinically validated biomarkers of ASD exist which can rapidly, accurately, and objectively identify autism during the first years of life and be used to support optimized treatment outcomes and advances in precision medicine. As such, the goal of the present study was to leverage both simple and computationally-advanced approaches to validate an eye-tracking measure of social attention preference, the GeoPref Test, among 1,863 ASD, delayed, or typical toddlers (12–48 months) referred from the community or general population via a primary care universal screening program. Toddlers participated in diagnostic and psychometric evaluations and the GeoPref Test: a 1-min movie containing side-by-side dynamic social and geometric images. Following testing, diagnosis was denoted as ASD, ASD features, LD, GDD, Other, typical sibling of ASD proband, or typical. Relative to other diagnostic groups, ASD toddlers exhibited the highest levels of visual attention towards geometric images and those with especially high fixation levels exhibited poor clinical profiles. Using the 69% fixation threshold, the GeoPref Test had 98% specificity, 17% sensitivity, 81% PPV, and 65% NPV. Sensitivity increased to 33% when saccades were included, with comparable validity across sex, ethnicity, or race. The GeoPref Test was also highly reliable up to 24 months following the initial test. Finally, fixation levels among twins concordant for ASD were significantly correlated, indicating that GeoPref Test performance may be genetically driven. As the GeoPref Test yields few false positives (~ 2%) and is equally valid across demographic categories, the current findings highlight the ability of the GeoPref Test to rapidly and accurately detect autism before the 2nd birthday in a subset of children and serve as a biomarker for a unique ASD subtype in clinical trials.

Autism spectrum disorder (ASD) begins during prenatal life^1,2^, yet most children do not receive a diagnosis and start treatment until 3–4 years later^3,4^. Although genetic, neural, metabolomic, and molecular systems are adversely impacted in ASD^2,5,6^, it is nevertheless detected and diagnosed using clinical judgement.

There has been a recent surge in research designed to discover biologically-based markers of ASD which can increase the pace of diagnosis, remove the requirement for highly-trained professionals, provide prognostic information, guide treatment plans, or be used as outcome measures in clinical trials^7^. Currently, only two ASD biomarkers are being considered for the FDA Biomarker Qualification Program^8,9^. However, they were established at “late” ages in children and therefore may not be generalizable to toddlers and infants, for whom biomarkers are of greatest utility. Moreover, these biomarkers only identify a subset of ASD children, indicating that additional biomarkers for other ASD subtypes are needed.

Dramatically reduced attention to social information is a key feature of ASD noted since its discovery in 1943^10^. Unsurprisingly, considerable effort has been leveraged to understand and quantify social visual attention abnormalities, most recently using eye-tracking^11–29^. Despite varying stimuli and participant age, a meta-analysis of 38 published eye-tracking studies indicated that subjects with ASD attend definitively less to social stimuli compared to typically developing individuals^30^.

Our previous eye-tracking work using a novel preferential looking paradigm known as the ‘GeoPref Test’ identified a subgroup of ASD toddlers with heightened visual attention towards geometric relative to social images^16,25^. The effect was robust with > 85% test–retest reliability, 86–100% positive predictive value (PPV), and 98% specificity, although sensitivity was consistently lower at ~ 20%^16,25^. ASD toddlers with a strong non-social preference also had higher symptom severity, worse language and cognitive ability^16^, weak functional connectivity between social-visual brain networks^31^, and worse school-age outcomes compared to social-preferring toddlers^20^. Because this work was conducted within the context of population-based screening^32,33^, one strength was the inclusion of non-ASD contrast groups which mimic the natural constituency found during routine pediatric practice. This is of considerable importance for the development of biomarkers with real-world utility. Weaknesses of the initial studies, however, include small sample sizes (i.e., 110 and 333), and a lack of rigorous methodology to examine predictive validity. We also failed to combine metrics to improve predictive accuracy, focusing instead on fixation levels alone. While other groups have utilized similar paradigms^13–15,26,27,34–36^ or a near identical version of the GeoPref Test^25^ and report similar results^19,37–48^, validation statistics are often unreported.

In addition to validation, establishing impactful biomarkers requires understanding for whom, and at what ages, the marker best applies. In fields outside of autism it is well known that biomarker efficacy differs by race, ethnicity, age, and sex^49,50^. With minor exception, eye-tracking studies within the autism field often have relatively few subjects^11,22,29,51^, yet sample sizes in the thousands are necessary for resolving demographic effects and establishing medical biomarkers^52–54^. Moreover, there is a need to examine associations between biomarkers and clinical profiles as a pathway towards individualized medicine. Finally, ASD biomarkers which are tuned for high specificity/low false positive rate are necessary for circumventing the financial burden and familial stress associated with false positive results.

There is also evidence for a genetic component of visual social preference^55–58^, but few studies have explored this using eye-tracking. We addressed this previously by comparing GeoPref Test performance in sibling pairs and found that among sibling pairs concordant for ASD, fixation to geometric images was strongly correlated^16^. In a different study, the time non-ASD monozygotic twins spent looking at eyes or mouth in an eye-tracking task was highly intercorrelated^59^. Such findings underscore the potential genetic basis of social visual attention patterns.

Given the necessity for well-developed, clinically relevant ASD biomarkers both for diagnostic purposes and use in clinical trials, the goal of the present study was to comprehensively validate an eye-tracking based biomarker in a large, diverse group of toddlers, producing the largest eye-tracking study of ASD and other delays to date. Here, we report ASD classification accuracy using both a simple, scalable approach associated with a single metric—percent fixation—that can be easily leveraged by non-researchers, as well as a more complex approach which utilizes machine learning algorithms to conduct tenfold validation and an independent replication set based on multiple eye-tracking metrics.

## Methods

### Participants

Subjects were referred through the community or via a population-based screening method known as *Get SET Early*^32,33^. Following screening at well baby check-ups using the CSBS IT-Checklist^60^, toddlers were referred to the University of California, San Diego Autism Center of Excellence for in-depth diagnostic evaluations and eye-tracking, and invited for repeat testing every ~ 12 months until age 3. Toddlers were assessed by licensed Ph.D.-level clinical psychologists blind to eye-tracking results using the Mullen Scales of Early Learning^61^, the Autism Diagnostic Observation Schedule^62^, and the Vineland Adaptive Behavior Scales^63^. Parents were given diagnostic feedback and toddlers referred for treatment as appropriate.

Of the 1,685 toddlers enrolled in the study, 266 (15.8%) were excluded largely due to compliance (See Supplemental Methods eFigure 1). The remaining 1,419 toddlers (mean age: 24.37 months, range: 12.00–49.11) were separated into diagnostic groups based on most recent diagnoses including ASD, ASD features (ASD-Feat), global developmental delay (GDD), language delay (LD), typically developing (TD), typical toddlers with an ASD sibling (TypSibASD), and Other (Table 1 and Supplemental Methods). This final sample is independent from our previous work^16,25^. To validate the GeoPref Test on the largest sample possible, secondary analyses included 444 toddlers from our previous work resulting in a cumulative sample of 1,863 toddlers. Among this sample are 11 monozygotic twins, 27 dizygotic twins, and 109 sibling pairs. Given the goal of early biomarker discovery, the first (i.e., youngest age) eye-tracking data collection was used in analyses.

**Table 1:** Summary of clinical characteristics for 1,863 toddlers who completed The GeoPref Test. Values are presented as means (standard deviations are noted in parentheses unless otherwise noted, and age range provided in brackets). ASD: Autism Spectrum Disorders, ASD-Feat: ASD Features, GDD: Global Developmental Delay, LD: Language Delay, TD: Typically Developing, TypSibASD: Typical Sibling of subject with ASD; ADOS: Autism Diagnostic Observation Scale, SA/CoSo Score: Social Affect/Communication Social Score, RRB Score: Restricted and Repetitive Behavior Score.

	Mean (SD)						
	ASD (N = 725)	ASD-Feat (N = 103)	GDD (N = 128)	LD (N = 198)	Other (N = 162)	TD (N = 487)	TypSibASD (N = 60)
**M/F**	563/162	85/18	98/30	149/49	113/49	295/192	28/32
**Age, months**	26.40 (8.25)	23.84 (9.17)	26.38 (9.81)	20.78 (7.44)	23.15 (9.31)	23.32 (9.17)	21.86 (8.79)
**Range**	[12–49]	[11–44]	[12–46]	[10–48]	[11–48]	[11–48]	[12–44]
**Ethnicity (%)**							
Hispanic or Latino	32.69%	29.13%	51.56%	42.93%	23.46%	19.71%	23.33%
Non-Hispanic or Latino	56.00%	63.11%	42.97%	50.00%	69.14%	71.25%	65.00%
Unknown/not reported	11.31%	7.77%	5.47%	7.07%	7.41%	9.03%	11.67%
**Race (%)**							
Caucasian	52.14%	66.99%	54.69%	53.03%	65.43%	67.15%	66.67%
American Indian/Alaska Native	0.83%	0.97%	0.78%	1.52%	1.23%	0.41%	0.00%
Asian	13.10%	5.83%	3.91%	8.08%	4.94%	8.01%	3.33%
Black/African American	2.48%	1.94%	1.56%	3.54%	1.85%	2.46%	3.33%
Pacific Islander/Native Hawaiian	0.83%	2.91%	2.34%	1.01%	1.23%	0.82%	1.67%
More than one race	9.79%	8.74%	10.94%	5.56%	13.58%	8.83%	8.33%
Not reported/unknown/other	20.83%	12.62%	25.78%	27.27%	11.73%	12.32%	16.67%
**Mullen (t score)**							
Visual reception	38.00 (13.04)	49.17 (12.31)	37.31 (11.82)	49.01 (10.13)	52.35 (11.87)	56.16 (9.68)	57.34 (9.34)
Fine motor	37.59 (16.23)	47.62 (12.05)	37.68 (12.39)	49.50 (10.31)	49.85 (11.84)	54.21 (9.14)	54.71 (8.86)
Receptive language	28.47 (12.78)	44.09 (13.06)	34.60 (10.79)	39.78 (11.86)	48.08 (13.15)	52.12 (10.78)	51.68 (10.42)
Expressive language	27.80 (13.03)	40.97 (12.17)	29.35 (11.23)	32.79 (9.59)	44.67 (12.04)	48.93 (10.72)	52.68 (10.44)
Early learning composite	69.01 (19.39)	91.66 (18.37)	71.19 (18.05)	86.20 (14.65)	96.83 (20.22)	105.83 (13.73)	108.63 (13.46)
**Vineland (standard score)**							
Communication	77.20 (13.90)	91.33 (12.41)	81.62 (13.32)	86.45 (9.66)	94.23 (12.98)	99.56 (11.38)	98.60 (15.28)
Daily living	84.65 (12.61)	92.38 (11.53)	87.59 (14.63)	95.27 (10.54)	95.23 (13.02)	99.67 (11.21)	100.67 (10.00)
Socialization	84.23 (11.97)	95.16 (9.72)	90.57 (12.92)	96.12 (10.89)	98.15 (11.73)	102.62 (9.44)	103.48 (8.82)
Motor skills	90.76 (10.67)	97.40 (10.63)	86.75 (16.67)	96.49 (8.81)	96.62 (11.95)	99.26 (9.63)	100.57 (8.80)
Adaptive behavior composite	81.08 (11.12)	92.50 (10.40)	84.50 (12.94)	91.37 (10.56)	95.07 (11.79)	99.99 (9.86)	101.10 (8.97)
**ADOS (module T or 1)**							
ADOS RRB score	4.36 (2.16)	2.22 (1.71)	1.39 (1.51)	0.96 (1.20)	1.13 (1.35)	0.52 (0.92)	0.57 (1.01)
ADOS Sa/CoSo tot score	13.24 (4.78)	6.94 (4.73)	4.72 (3.65)	3.82 (2.86)	4.06 (3.57)	2.87 (2.59)	2.05 (1.87)
ADOS total score	17.60 (5.87)	9.16 (5.37)	6.10 (4.20)	4.77 (3.18)	5.20 (4.16)	3.39 (2.96)	2.62 (2.24)

### Eye-tracking apparatus, stimuli, and procedures

**Figure 1:**
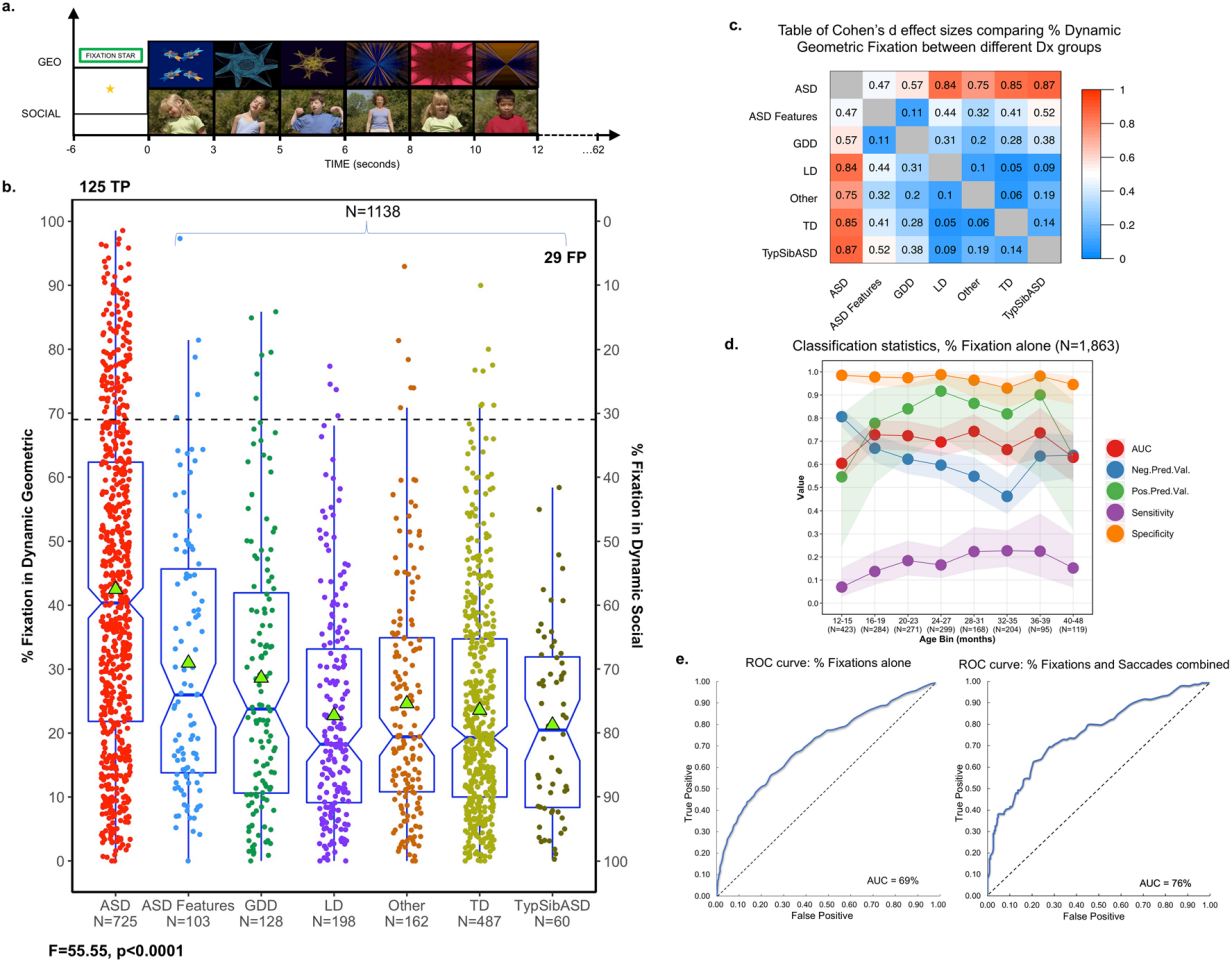
Validation of visual attention patterns among a combined sample of 1,863 toddlers of varying developmental types. (**a**) Sample images from the GeoPref eye-tracking test across the first 12 s. Social images courtesy of Gaiam Americas Inc., Copyright 2003, Gaiam Americas, Inc. To standardize initial fixations across toddlers, testing was preceded by a fixation star which flashed for 6.23 s. The entire length of the test was 62.23 s. (**b**) In the current study, 444 toddlers from a previous analysis^16^ were combined with a new, independent sample of 1,419 toddlers in order to increase power and provide the largest dataset possible for subsequent analyses. As such, scatterplot demonstrates percent fixation to dynamic geometric images (DGI) or dynamic social images (DSI) for the combined sample of 1,863 toddlers. Percent fixation was defined as fixation duration within geometric or social areas of interest divided by total fixation duration to the entire video. Dashed lines depict threshold for 69% fixation. Green triangles indicate average percent fixation. Notched boxplots show median, range, and first/third quartiles. Notch ranges indicate 95% confidence intervals around the median. TP: true positive, FP: false positive. F statistic pictured was obtained from a one-way ANOVA conducted to compare percent DGI fixation across diagnostic groups. (**c**) Effect size matrix of pairwise comparisons of average percent fixation to DGI for toddlers of varying developmental types. Warmer colors indicate larger effect sizes and cooler colors indicate smaller effect sizes. (**d**) Validation statistics for classification of toddlers as ASD vs. non-ASD using a 69% fixation threshold after grouping toddlers into 4-month age bins. Toddlers > 40 months were binned together given the small sample size of toddlers beyond this age. (**e**) ROC curves showing classification performance (ASD vs. non-ASD) among 20% of toddlers which make up an independent, hold-out test using left, percent DGI fixation alone (threshold: 61.3%), or right, multiple eye-tracking metrics (percent fixation, threshold: 61.3% and saccades/second, threshold: 2.29 saccades/second). ASD: Autism Spectrum Disorders, ASD-Feat: ASD Features, GDD: Global Developmental Delay, LD: Language Delay, TD: Typically Developing, TypSibASD: Typical Sibling of subject with ASD, AUC: Area under the curve, Neg. Pred. Val.: Negative Predictive Value, Pos. Pred. Val.: Positive Predictive Value.

Eye-gaze data was collected using the Tobii T120 (Tobii, Stockholm, Sweden; www.tobii.com; 60 Hz sampling rate; 1280 × 1024) while toddlers watched ‘The GeoPref Test’ (62.22 s), which consisted of two rectangular areas of interest (AOIs, 525 × 363 pixels) each containing dynamic geometric (DGI) or social images (DSI; social images used with permission from Gaiam Americas Inc., Copyright 2003, Gaiam Americas, Inc.), identical to stimuli used in our previous work^16,17,25^ (Fig. 1A; Supplemental Methods eFigure 2). To control for biases due to spatial location, side of stimulus presentation varied across subjects.

To ensure that only the toddler’s gaze was tracked and free from parent influence, standardized instructions were read to parents. A five-point calibration was then performed using animated cartoon ducks with sounds, and data was only used if calibration results, determined via graphical output and verified via screenshots, fell within manufacturer-reported parameters (accuracy, 0.5 degrees^64^). For a subset of toddlers, a flashing star with chime appeared for 6.23 s prior to the start of the experiment to ensure toddlers fixated to the screen.

Data was processed using Tobii Studio (Tobii Fixation Filter, velocity threshold: 35 ms/window). Total fixation duration, fixation count within each AOI, and fixation duration within each AOI were exported and analyzed offline. Percent fixation duration/AOI was computed by dividing the total fixation duration within an AOI by the fixation duration across the entire video. N-1 total fixations/total fixation duration was used to calculate saccades/sec within each AOI.

### Defining ASD subgroups

For comparability with our earlier work and to tune the test towards a low false positive rate, 69% fixation to either DGI or DSI was used to distinguish between ASD toddlers who strongly preferred to visually fixate on geometric images (ASD_Geo_) or social images (ASD_Soc_)^16,25^. Given that experience-dependent mechanisms may differentially impact toddlers with distinct attention patterns, stratifying ASD toddlers in this way may have relevance for understanding long term outcomes among subtypes identified through eye-tracking. ASD toddlers who lacked a strong preference for either stimulus (i.e. they fixated on DGI for 32–68% of the time) were categorized as ASD Middle responders (ASD_Mid_).

### Statistical analyses

All analyses were conducted using R and relevant packages (e.g., pROC, ROCR^65–74^). Results are reported using associated 95% confidence intervals (CI) and effect sizes as appropriate.

### Visual attention preference stratified by diagnosis and correlation with clinical measures

One-way ANOVA was performed to compare percent fixation towards DGI across diagnostic groups. Follow-up pairwise comparisons of group means were conducted using Tukey’s HSD.

Linear regression with percent DGI fixation as the outcome variable and sex, age, and diagnosis as predictor variables was used to examine associations between demographics and DGI fixation levels. Pearson’s correlations were conducted to examine relationships between DGI fixation and performance on clinical measures.

Across ASD subgroups, performance on clinical measures and associated subscales was assessed via one-way ANCOVA with age and sex as covariates. Subgroup-level differences were assessed using Tukey’s HSD.

### Classification accuracy: traditional approaches

To determine whether validation statistics for the current sample were comparable to those observed in our previous work^16,25^, and to tune the test in favor of a low false-positive rate, the same 69% fixation threshold was applied here to the full cohort to compute Receiver Operating Characteristic (ROC) curves, an Area Under the Curve (AUC) statistic, sensitivity, specificity, positive predictive value (PPV), and negative predictive value (NPV).

Validation statistics were also computed after stratifying toddlers based on age bins (4-months), ethnicity, race, and sex.

### Saccade profiles within ASD

Our previous work indicated that ASD_Geo_ toddlers exhibited significantly fewer saccades when viewing DGI and significantly more when viewing DSI relative to other ASD toddlers^16,17,25^. To determine whether these patterns were also present in this larger sample, a one-way ANCOVA with age and sex as covariates and a follow up Tukey’s HSD test was used.

### Classification accuracy: cross validation with independent hold out set

Rigor, generalizability, and diagnostic accuracy of the GeoPref Test were examined using a tenfold cross-validation approach conducted on 80% of the cohort who were randomly selected to be included in the analysis. Ten-fold cross-validation is a widely used method for parameter tuning and threshold selection in statistical learning and classification problems^75^. Average performance statistics for all 10 validations were averaged and reported, and then used to classify the remaining 20% of toddlers which made up an independent, hold-out test set. See Supplemental Methods.

Classification sensitivity enhancement was also examined using tenfold cross-validation following the inclusion of both percent fixation and a second eye-tracking metric, number of saccades per second within DSI, as variables of interest.

### Test–retest reliability

Additional eye-tracking sessions (not used in aforementioned analyses) occurred in 535 toddlers (mean age: 19.48 ± 6.18 months) within 1 to 25 + months of their first eye-tracking test. After grouping toddlers based on interval length (immediate: 0–1 month, n = 39; short term: 2–6 months, n = 100; intermediate term: 7–12 months, n = 211; long term: 13–24 months, n = 163; and very long term: > 25 months, n = 22), intraclass correlations and paired t-tests were used to examine differences in percent DGI fixation between tests.

### Development of visual social attention preference across the first years of life

To better understand the trajectory of typical versus atypical visual attention patterns, Pearson’s correlations comparing age and percent DSI/DGI fixation were conducted for all diagnostic groups. The impact of age on eye-tracking performance was further examined using linear regression with percent fixation on DSI as the dependent variable, and diagnosis, age, and diagnosis x age as predictor variables.

### Examination of genetic underpinnings of visual social attention

Intraclass correlations were used to determine the concordance among social visual attention patterns between siblings, twin pairs and a random pairing of 850 unrelated subjects.

### Ethical approval statement

This study was approved by the Institutional Review Board at the University of California, San Diego (IRB #181,652, #172,066, #081,722, #041,715, #140,673, #130,352, #110,049, #070,229) and performed in accordance with the UCSD Human Research Protections Program guidelines. Prior to data collection, informed consent was obtained from all subjects and/or their legal guardians for study participation. GeoPref Test social images are copyrighted by Gaiam Americas Inc. and were used with permission.

## Results

### Visual attention preference stratified by diagnosis and correlation with clinical measures

Similar to our previous study^25^, results from the independent sample of 1,419 toddlers indicated significant differences in the amount of time a toddler fixated on DGI based on diagnostic group membership (F(6,1,412) = 43.74, p < 0.0001). There were no differences in terms of data distribution (Kolmogorov–Smirnov Z Test, D = 0.042, p = n.s.) or effect sizes between the new independent sample and our prior smaller sample of 444^25^ toddlers, and thus both were combined to increase power and provide the largest dataset possible for subsequent analyses (N = 1,863). See Supplemental Results and eFigure 3 for the independent sample data. Within the combined sample of 1,863, toddlers with ASD exhibited the highest percent fixation to DGI compared to all other toddler types (ASD 95% CI [40.67, 44.30] vs. ASD-Feat 95% CI [26.88, 35.03], mean difference: 11.53 ± 4.03, p < 0.0001, d = 0.47 95% CI [0.26, 0.68]; ASD vs. GDD 95% CI [24.86, 32.35], mean difference: 13.88 ± 3.46, p < 0.0001, d = 0.57 95% CI [0.38, 0.76]; ASD vs. LD 95% CI [20.30, 25.19], mean difference: 19.74 ± 7.44, p < 0.0001, d = 0.84 95% CI [0.68, 1.00]; ASD vs. Other 95% CI [21.70, 27.51], mean difference: 17.88 ± 6.15, p < 0.0001, d = 0.75 95% CI [0.57, 0.92]; ASD vs. TD 95% CI [22.03, 25.12], mean difference: 18.91 ± 7.54, p < 0.0001, d = 0.85 95% CI [0.73, 0.97]; ASD vs. TypSibASD 95% CI [17.50, 25.04], mean difference: 21.21 ± 10.29, p < 0.0001, d = 0.87 95% CI [0.61, 1.14]). Preference for DGI was also high for the ASD-Feat group (ASD-Feat vs. TD mean difference: 7.38 ± 3.51, p < 0.05, d = 0.41 95% CI [0.20, 0.62]; ASD-Feat vs. LD mean difference: 8.21 ± 3.41, p < 0.05, d = 0.44 95% CI [0.20, 0.68]). TD, LD, GDD, TypSibASD, and toddlers categorized as Other exhibited a stronger preference for DSI, and preference strength was comparable between groups. See Fig. 1. There were no differences in percent fixation levels when data was stratified by sex, although small differences were associated with ethnicity and race. See Supplemental Results eFigure 4–6.

Since diagnosis and age were significant predictors of DGI fixation (overall R^2^ = 0.21, F(7,1855) = 72.27, p < 0.0001), correlation coefficients were computed for each diagnostic group separately to determine relationships between DGI fixation and clinical symptoms. For toddlers with ASD, percent DGI fixation was significantly correlated with all clinical measures and all associated subscales. In contrast, apart from the visuospatial subscale on the Mullen, there were no significant relationships found between DGI fixation and clinical profiles within the TD or TypSibASD toddlers. See Supplemental Results eTable 1.

**Figure 2:**
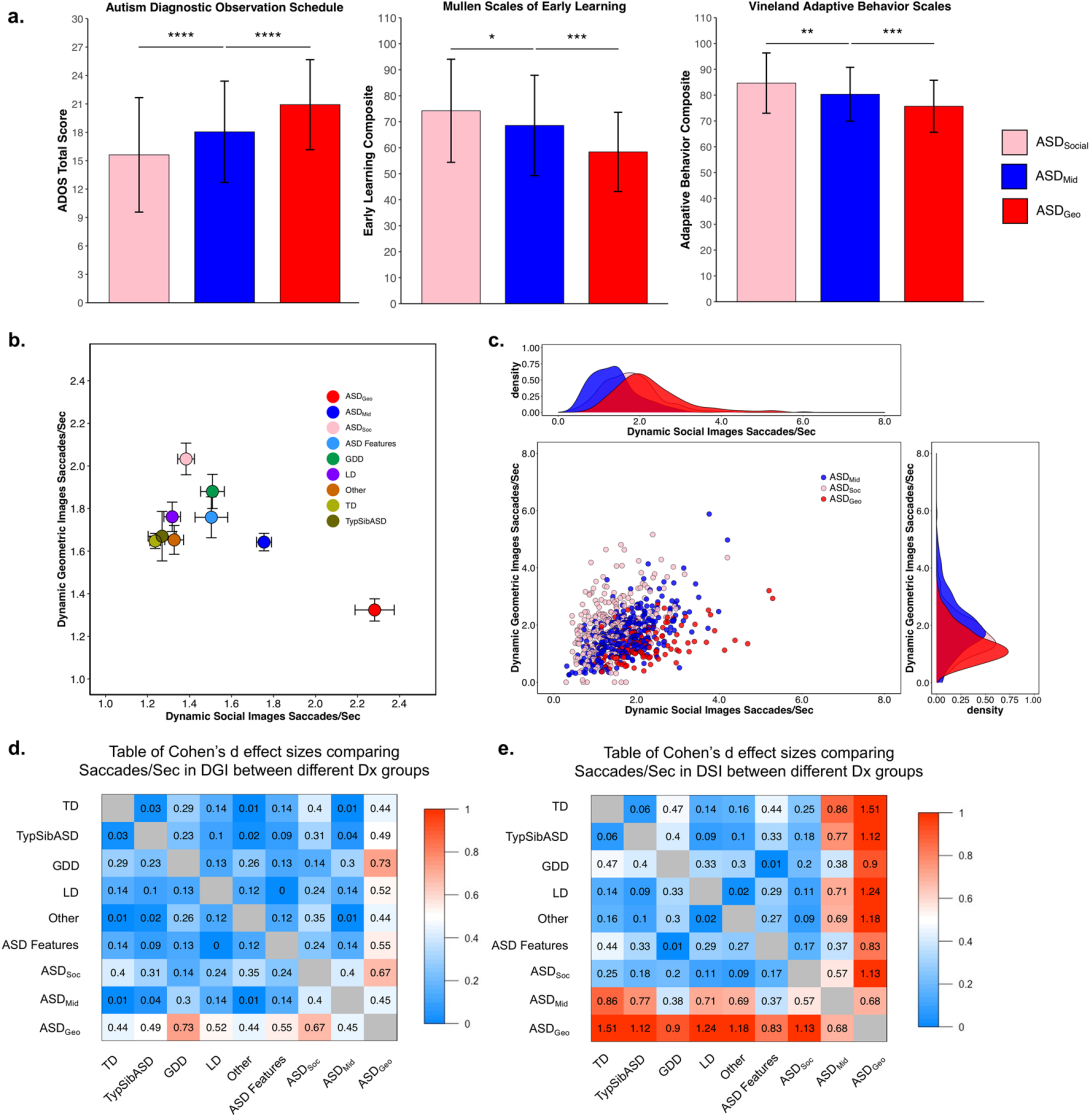
Differences in clinical and saccade profiles across ASD subgroups. (**a**) Bar graphs (mean ± SD) demonstrating clinical scores obtained from ASD toddlers with varying levels of preference for dynamic geometric images (DGI). ASD toddlers with a strong preference for DGI (≥ 69% fixation to DGI) were designated as ASD_Geo_. ASD toddlers with a strong preference for dynamic social images (DSI) (≥ 69% fixation to DSI) were designated as ASD_Soc_. Toddlers who lacked a strong preference for either stimulus were designated as ASD_Mid_ toddlers. (**b**) Scatterplot showing average saccades/sec across diagnostic groups (mean ± SEM) to dynamic geometric images (DGI, y-axis) or dynamic social images (DSI, x-axis). (**c**) Scatterplot showing saccades/sec to DGI (y-axis) or DSI (x-axis) for all ASD subgroups. Density plots demonstrate distribution of individual saccade patterns to DGI or DSI. (**d**,**e**) Cohen’s d effect sizes associated with pairwise comparisons of average saccades/sec to DGI or DSI. ASD: Autism Spectrum Disorders, ASD-Feat: ASD Features, GDD: Global Developmental Delay, LD: Language Delay, TD: Typically Developing; TypSibASD: Typical Sibling of subject with ASD.

Among the 3 ASD subtypes (see Methods), significant differences in symptom severity (ADOS total score F(2,707) = 37.21, p < 0.0001), cognitive ability (Mullen Early Learning Composite F(2,692) = 21.04, p < 0.0001), and adaptive behavior (Vineland Adaptive Behavior Composite (F(2,707) = 20.84, p < 0.0001) were observed, suggesting the possibility of unique underlying biological profiles. The largest differences were between toddlers who strongly preferred geometric (ASD_Geo_) and those that strongly preferred social (ASD_Social_) images. See Fig. 2a.

### Classification accuracy: traditional approaches

ROC curves leveraging the full dataset yielded 98% specificity, 17% sensitivity, 81% PPV, and 65% NPV when the 69% fixation threshold was used. See Supplemental Results eTable 2. These values are nearly identical to validation statistics computed in previous work using the same fixation threshold^16^. These results indicate that even among a large population of toddlers of various developmental types, the GeoPref Test accurately distinguishes ASD from non-ASD toddlers, with relatively few false positives (i.e., 2%), which is a key criterion for biomarker tests^76^.

Age-binned data show that while specificity remains high throughout development (> 90%), sensitivity is consistently low. Negative predictive value is highest at 12 months and lowest by 32 months, while peak positive predictive value is achieved by 24 months. See Fig. 1d.

We additionally examined classification statistics after stratification using demographic factors and found that the GeoPref Test performs similarly across sex, ethnic and racial groups. See Supplemental Results eTable 3.

### Saccade profiles within ASD

In our previous work, we demonstrated that ASD_Geo_ toddlers exhibited significantly fewer saccades/second when viewing DGI, but greater saccade rates when viewing non-preferred social images, in contrast to ASD_Soc_ toddlers who had near-typical saccade patterns^16^. This was replicated in the current large sample when saccades/second was examined within DGI (F(8,1847) = 9.65, p < 0.0001) or DSI (F(8,1847) = 33.24, p < 0.0001) images. All planned comparisons between ASD_Geo_ and other diagnostic groups were significant, with the largest effect sizes found between ASD_Geo_ and TD toddlers. See Fig. 2b-e.

### Classification accuracy: cross validation with independent hold out set

To enhance the rigor of the GeoPref Test, we next performed tenfold cross validation to determine appropriate DGI fixation thresholds for computing validation statistics. Using this method, the ideal fixation threshold was 61.3%, which yields 95% specificity, 23% sensitivity, 76% PPV, 66% NPV, and 67% accuracy. When this same threshold was applied to an independent, hold-out test set, the GeoPref Test had 96% specificity, 33% sensitivity, 84% PPV, 69% NPV, and 71% accuracy.

Despite high specificity using the 69% fixation threshold^16,25^ or the 61% fixation threshold obtained from tenfold cross validation, sensitivity remained low. However, combining eye-tracking measures, including saccades per second within DSI (optimal threshold from tenfold cross validation was 2.29 saccades/sec), and percent DGI fixation (61.3% fixation threshold), enhanced GeoPref Test sensitivity to 33.3%, with little impact on specificity (95.2%), PPV (81.4%) or NPV (71.2%) for the independent, hold-out test set. See Fig. 1e.

### Test–retest reliability

A key component of biomarker validation research is characterizing the stability of test performance across repeated measurements. Five hundred and thirty-five toddlers participated in repeat eye tracking. High levels of reliability were observed for more immediate, 0–1 month retests (intraclass correlation coefficient = 0.76, p < 0.0001; paired samples t-test t(75.32) = -0.72, p > 0.05). Longer interval retests were still reliable, although correlation strength was reduced. This result is expected as the GeoPref Test was created for assessing ASD during the toddler period, during which age-related visual attention changes are expected. See Supplemental Results eTable 4.

### Developmental trajectory of visual social attention preference across the first years of life

**Figure 3:**
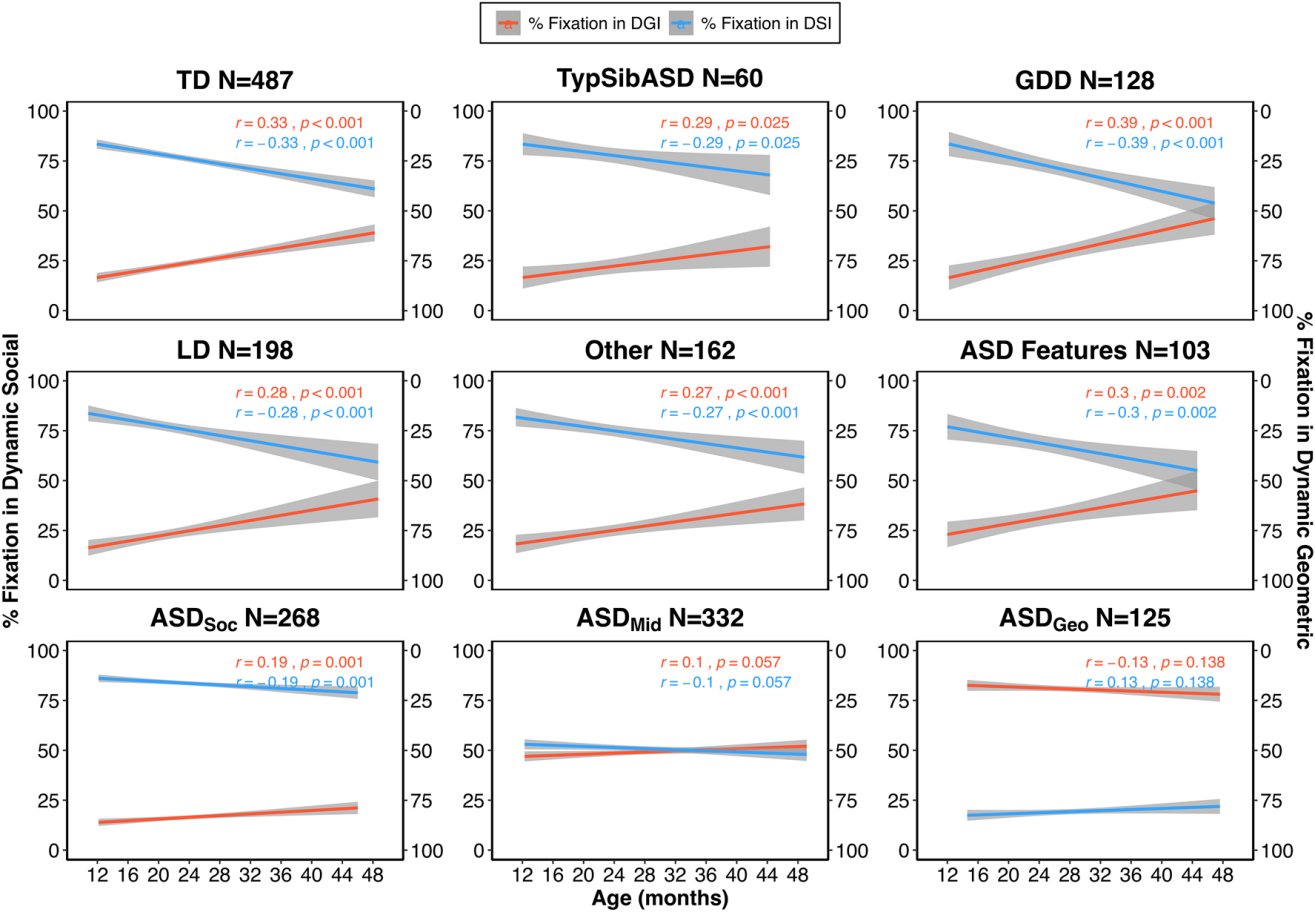
Developmental trajectories of social and non-social (geometric) attention distinguish ASD toddlers from toddlers of other developmental types. Best fit lines depicting developmental trajectories of percent fixation to social images (blue) or non-social images (red) for toddlers of different developmental types and associated Pearson’s r and p-values. ASD toddlers were again split into the three ASD subgroups based on visual attention preference for social (ASD_Soc_) or non-social stimuli (ASD_Geo_). ASD_Mid_ toddlers did not exhibit a strong preference for social or non-social stimuli. ASD: Autism Spectrum Disorders, ASD-Feat: ASD Features, GDD: Global Developmental Delay, LD: Language Delay, TD: Typically Developing, TypSibASD: Typical Sibling of subject with ASD.

Examination of the correlation between social and non-social fixation levels and age across all diagnostic groups and ASD subtypes revealed an interesting trend: both ASD toddlers with the social profile (ASD_Soc_) and other non-ASD toddlers significantly decreased their interest in social images with age, alongside a concomitant increase in interest in non-social images. In contrast, toddlers with the geometric profile (ASD_Geo_) as well as those that fell into the middle category (ASD_Mid_) showed no age-related changes. See Fig. 3. Follow-up linear regression analyses and pairwise comparisons of beta coefficients confirm this result (overall fit: F(17,1845) = 151.5, R^2^ = 0.58; ASD_Geo_ vs. TD p < 0.001, ASD_Geo_ vs. TypSibASD p < 0.05, ASD_Geo_ vs. GDD p < 0.0001, ASD_Geo_ vs. LD p < 0.001, ASD_Geo_ vs. Other p < 0.01, ASD_Geo_ vs. ASD-Feat p < 0.001).

### Sibling and twin correlations highlight the genetic underpinnings of visual social attention in ASD

**Figure 4:**
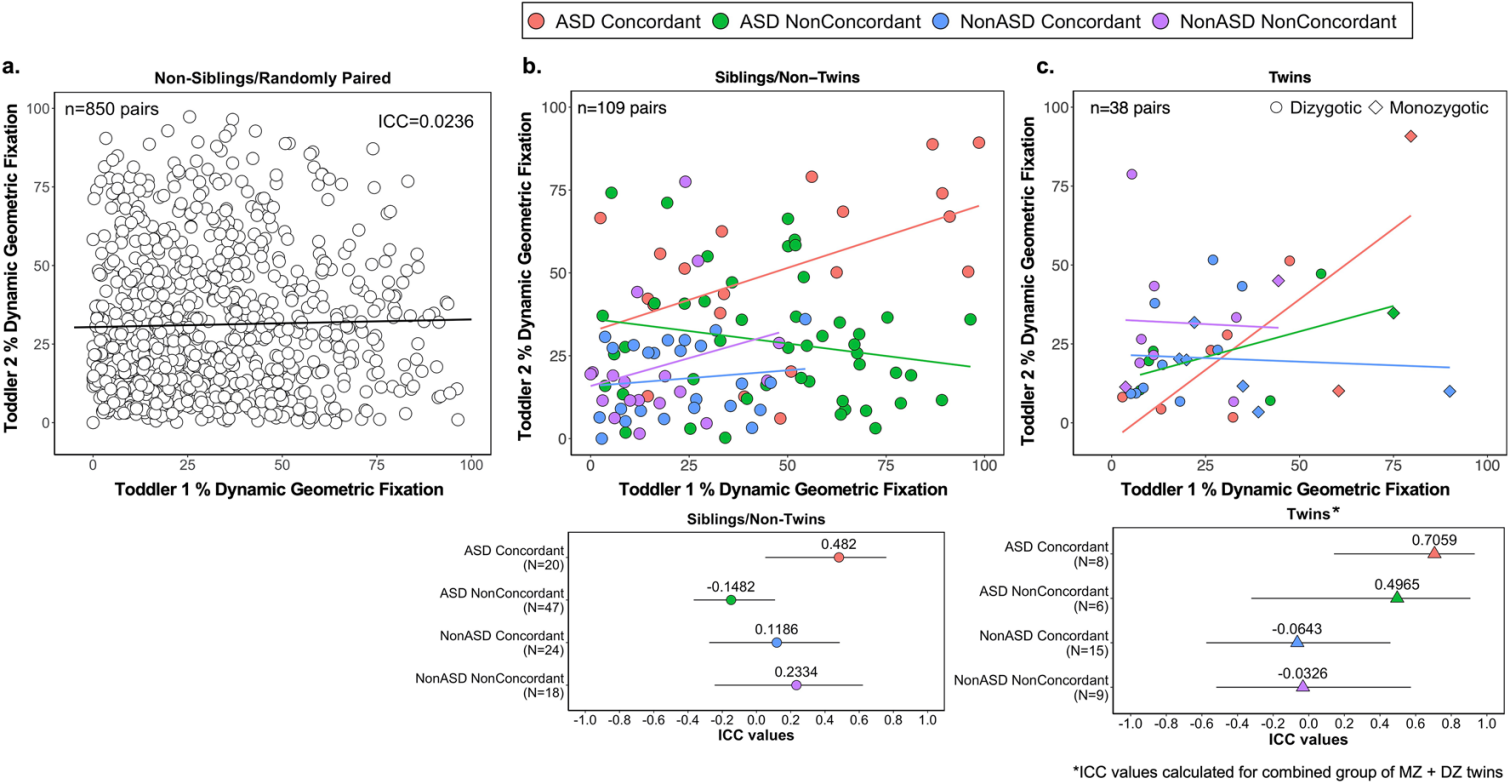
Eye-tracking among siblings and monozygotic and dizygotic twins highlights the genetic basis of the GeoPref Test. Scatterplots comparing percent fixation to dynamic geometric images (DGI) among (**a**) non-sibling/randomly paired toddlers, (**b**) sibling/non-twin toddler pairs, and (**c**) twin toddler pairs. Intraclass correlation coefficients are also shown in (**a**) or plotted, along with the 95% confidence intervals, based on concordance for ASD (**b**,**c**, bottom). ASD Concordant: both siblings/twins received a diagnosis of ASD. ASD NonConcordant: One sibling/twin received an ASD diagnosis while the other received a non-ASD diagnosis. NonASD Concordant: both siblings/twins received the same diagnosis but were non-ASD. NonASD NonConcordant: Each sibling/twin had a different, non-ASD diagnosis.

The current sample size allows us to compare DGI fixation among a larger group of siblings than our previous work^16^. ASD Concordant pairs of siblings or twins exhibited the highest ICC values compared to ASD NonConcordant pairs, NonASD Concordant pairs, NonASD NonConcordant pairs, and randomly paired subjects (Fig. 4). These findings highlight the sensitivity of the GeoPref Test for detecting a genetically driven subtype of ASD.

## Discussion

ASD begins during pregnancy^2,77–80^, and thus it is not surprising that parents become aware of developmental delays within the first months of their child’s life. Despite this, age at first diagnosis has remained stable at around ~ 52 months across the past decade^3,4,81^. To the degree that early identification and interventions are beneficial, alternatives to the long diagnostic journey are needed. Biomarkers offer one such alternative.

Using the largest eye-tracking sample to date with 1,863 toddlers who received both eye-tracking and a diagnostic evaluation by licensed psychologists, here we present comprehensive validation of an eye-tracking biomarker of an autism spectrum disorder subtype that quantifies a toddler’s attention to non-social images that is highly replicable and reliable. Importantly, participating toddlers were largely first identified through universal screening, underscoring the notion that eye-tracking may be an excellent 2^nd^ tier screen or diagnostic tool.

The GeoPref Test was examined in multiple ways. First, as a simple tool by examining only percent fixation on geometric images, which requires little to no computational sophistication, supporting use by clinicians and researchers alike. Moreover, the user can determine the specificity rate they prefer, as illustrated in the ROC table (See Supplemental Results eTable 2), and select the associated fixation cut off level. For example, in our study, 69% fixation was selected, which results in a 2% false positive rate. Tuning biomarker tests towards a very low false positive rate may be particularly important for disorders of infancy to avoid unnecessary parental stress associated with false positive results. Second, using a more rigorous machine learning, tenfold cross-validation approach with potentially more generalizable results than standard approaches, the present study found 95% specificity and 23% sensitivity. Incorporating an additional measure, saccades per second while viewing social images, increased the sensitivity to 33% while maintaining levels of specificity at 95%. This supports the notion that combining eye-tracking paradigms and/or metrics can bolster classification accuracy^34,82^.

Although the GeoPref Test has a low false positive rate and exceptional specificity, sensitivity was modest. Given the considerable heterogeneity inherent in ASD^31,83–86^, and the fact that several studies highlight the likelihood of specific subtypes in ASD, this is not surprising^87,88^. Toddlers who demonstrate reduced levels of social visual attention as measured by the GeoPref Test may indeed represent a unique biological subtype. In comparison to toddlers who strongly preferred social stimuli, average symptom severity among ASD_Geo_ toddlers as indexed by the ADOS was 5.6 points higher, while levels of cognition and adaptive behavior were 15.8 and 9.0 points lower, respectively. A unique imaging study of toddlers who received both eye-tracking and brain imaging revealed that ASD_Geo_ toddlers exhibited unusually low levels of connectivity between areas classically associated with the ‘social brain’ (e.g., cingulate) and visual cortex^31^, and a separate study found strong correlations between social visual attention levels and language/visual cortex connectivity in this subgroup^89^. Collectively, these studies suggest that ASD_Geo_ toddlers are more symptomatic with a unique genetic profile that drives abnormal neural development, particularly as it relates to connectivity with visual cortex. Future studies which incorporate additional eye-tracking paradigms tailored to quantify other features and/or subtypes^12,13,82,90–92^ in conjunction with the GeoPref Test will likely capture more of the variance associated with ASD and improve test sensitivity.

Although there is extensive evidence supporting the notion of reduced social attention in ASD^30^, it is not possible to definitively conclude whether the present findings reflect a failure of social attention, or a difference in visual preference driven by sensory issues common in ASD^93,94^. For instance, the geometric patterns are per pixel more dynamic than the social videos and involve more color change. The geometric patterns may also be more unpredictable than the social scene. There is evidence that altered sensory processing may be a direct or indirect driver of social attention and/or orienting and higher order social ability among ASD toddlers^95–97^, indicating that the GeoPref Test may be a correlative measure of ability in either or both domains. Future characterization of sensory profiles across groups may help to deepen our understanding of drivers of visual attention patterns in ASD in general, and the ASD_Geo_ subtype more specifically.

Although our test was specifically tuned for the 12–48-month age range, an important consideration is whether biomarkers have comparable efficacy across target ages. Here we found good psychometric properties between 12 and 39 months, with decline in accuracy starting around 40 months, which may be attributed to age-related changes in social preference in typically developing infants^98–104^. This process, likely driven by frontal cortex synapse proliferation followed by selective pruning across the first years of life^105^, affords the child greater curiosity, control, and preference for novelty as they age^106,107^. Indeed, TD and non-ASD delayed toddlers demonstrated a simultaneous reduction in social preference and increase in geometric preference (i.e., “novelty”) across age. While ASD_Soc_ toddlers showed a profile almost identical to typically developing toddlers, ASD_Geo_ toddlers did not. To the degree that eye tracking performance has external validity and can serve as a proxy for real-world social engagement, results from the GeoPref Test may be useful as a prognostic metric. Indeed, one study noted that toddlers with ASD who preferred social images had better symptom profiles at school age than those that preferred geometric images^20^.

The psychometric properties of the GeoPref Test were also comparable across demographic categories, which may have been bolstered by the fact that it is a visual-only (i.e., no sound) test, potentially circumventing biases associated with language or culture^108–111^. Although the gap between first age of diagnosis and treatment access is narrowing between Caucasian and non-Caucasian children^3^, racial and ethnic inequities persist^3,112^. Females are also more likely to be diagnosed at older ages^108,109,113^. Such findings underscore the need for the development and implementation of culture-free, objective tools which support equal access to early diagnosis and treatment^114^.

The high intercorrelation of geometric fixation levels between ASD siblings and twins in our study suggests that eye-tracking biomarkers may be essential for early identification of genetic forms of ASD. A recent study noted a high intercorrelation in visual social attention towards the mouth and eyes among non-ASD twins, but not in unrelated children^59^. These results suggest that eye-tracking measures of social visual attention can be driven by ASD-related genetic variance or by genes which drive social attention. These genes may not be mutually exclusive. For instance, infants homozygous for the CD38 risk allele exhibited less gaze to happy eyes compared to infants heterozygous or homozygous for the non-risk allele^56^. Relatedly, CD38 risk allele expression is associated with higher ASD symptom severity^115^. Future work examining genetic profiles which drive ASD subtypes will help clarify the impact of ASD and other genetic factors on eye-tracking performance.

The large sample size in the current study, and the strong psychometric properties across age, sex, race and ethnic groups generates confidence that the GeoPref eye-tracking Test has value as both a clinical and research tool. Moreover, our sample included multiple non-ASD contrast groups (e.g., LD, GDD) which mimics natural pediatric practice. Importantly, performance on clinical measures in the current study correlated with geometric fixation levels, suggesting that the GeoPref Test can directly index a core ASD phenotype, which is relevant for measuring clinical improvements in clinical trials. Eye-tracking metrics outlined in the current study and others like it may be key in ensuring that severely impacted toddlers receive early diagnosis and treatment access, and promote biotherapeutic and behavioral treatment development, which can contribute to better outcomes and quality of life.

## Supplementary Information

Supplementary Information.

## Data Availability

The datasets generated and analyzed during this study are available from the corresponding author on reasonable request.
