# Supplementary material for: Large scale validation of an early-age eye-tracking biomarker of an autism spectrum disorder subtype
Source: Sci Rep. 2022 Mar 11;12:4253. doi: 10.1038/s41598-022-08102-6 (PMC8917231; doi:10.1038/s41598-022-08102-6)
Supplement: Supplementary file 1 — Supplementary Information. [file 41598_2022_8102_MOESM1_ESM.pdf]

SUPPLEMENTARY MATERIALS

Large scale validation of an early-age eye-tracking biomarker of an autism spectrum disorder subtype

Teresa H. Wen, Ph.D.[1], Amanda Cheng, B.S.[1], Charlene Andreason, M.A.[1], Javad Zahiri, Ph.D.[1], Yaqiong Xiao, Ph.D.[1], Ronghui Xu, Ph.D.[3], Bokan Bao, M.S.[1,2], Eric Courchesne, Ph.D.[1], Cynthia Carter Barnes, Ph.D.[1], Steven J. Arias, Ph.D.[1] & Karen Pierce, Ph.D.[1]

Affiliations:
[1]Autism Center of Excellence, Department of Neurosciences, University of California, San Diego, La Jolla, CA, USA
[2]Department of Bioinformatics and Systems Biology, University of California, San Diego, La Jolla, CA, USA
[3]Herbert Wertheim School of Public Health and Department of Mathematics, University of California, San Diego, La Jolla, CA, USA

Supplemental Methods – Diagnostic Criteria
Supplemental Methods – Classification Accuracy and Prediction Performance Evaluation Using Cross Validation
Supplemental Methods – Summary of Excluded Eye-Tracking Data, eFigure 1
Supplemental Methods – Dynamic Geometric and Social Stimuli Which Make Up the 'GeoPref Test', eFigure 2
Supplemental Results – Visual Attention Patterns Among an Independent Sample of 1,419 Toddlers, eFigure 3
Supplemental Results – Visual Attention Patterns Stratified by Ethnicity, Race, and Sex, eFigure 4, 5, & 6
Supplemental Results – Relationships Between DGI Fixation Levels and Clinical Measures Among the Full Set of 1,863 Toddlers, eTable 1
Supplemental Results – Validation Statistics as a Function of DGI Fixation Threshold, eTable 2
Supplemental Results – Validation of GeoPref Test Performance Stratified by Ethnicity, Race, and Sex, eTable 3
Supplemental Results – Reliability of GeoPref Test Performance in Toddlers with Multiple Eye-Tracking Sessions, eTable 4

Supplemental Methods

Diagnostic Criteria

A toddler was designated in each of the following diagnostic categories based on the following criteria: *Autism Spectrum Disorder (ASD)* – scored within the range of concern on the ADOS and was considered ASD based on DSM 5 criteria and clinical judgment. *ASD Features (ASD-Feat)* – showed signs of autism and may have an elevated ADOS score but did not meet full criteria for ASD. *Global Developmental Delay (GDD)* – 1 standard deviation below expected values on two or more areas of the Mullen with at least one of those areas outside of the verbal scales. *Language Delay (LD)* – 1 standard deviation below expected values on either or both the receptive and expressive subtests on the Mullen Scales of Early Learning. *Other* – showed developmental issue not captured in any of the aforementioned categories including motor delay, social emotional delay, attention deficit and speech articulation impediment. Toddlers were determined to be *Typically Developing (TD)* if they fell within the normal range on all clinical assessments and *TypSibASD* if they also had sibling with ASD.

Classification Accuracy and Prediction Performance Evaluation Using Cross Validation

To perform 10-fold cross validation the data was randomly partitioned into 10 non-overlapping subsets. Nine subsets served as the training subset, while the 10th subset acted as the validation subset. Given challenges inherent in early diagnosis wherein children across a range of delays and disorders may have overlapping symptom profiles, ideally biomarkers should be tuned to have a low false positive rate and high levels of specificity. As such, 95% specificity was used as the criteria for determining an appropriate DGI fixation threshold for an ASD diagnosis. As such, a fixation threshold which yielded optimal specificity was selected and used to measure performance on the validation set. This process was repeated 10 times such that all 10 subsets served as the test subset at least once.

Summary of Excluded Eye-Tracking Data

Among our initial independent sample of 1,685 toddlers, 266 were excluded from analyses due to a variety of reasons, show below in eFigure 1.

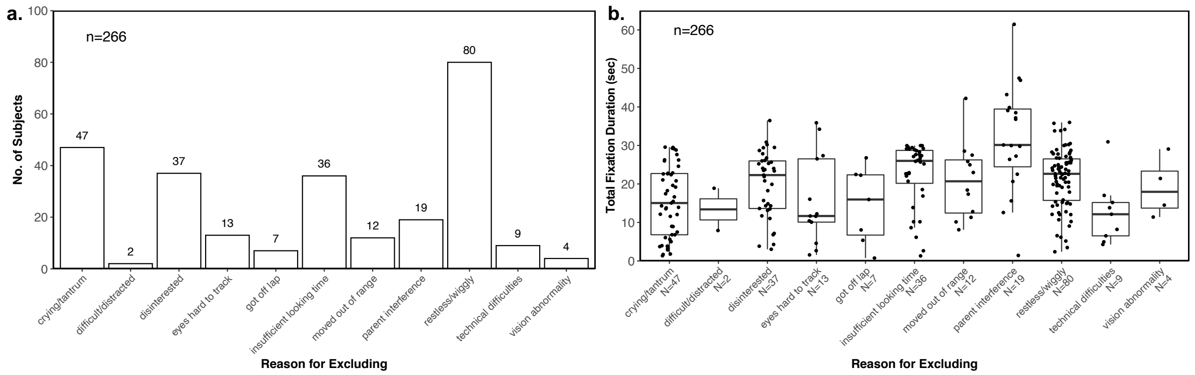

eFigure 1. Summary of excluded eye-tracking data. Left, Bar graph showing the number of subjects excluded due to a variety of reasons including insufficient looking time, parent interference, and restlessness/wiggling. Toddlers were also excluded for insufficient looking time if their total fixation duration to either dynamic geometric or dynamic social images was less that 50% of total looking time. **Right,** Scatterplots showing total fixation duration for excluded subjects. Boxplots show median and first/third quartiles.

Dynamic Geometric and Social Stimuli Which Make Up the 'GeoPref Test'

The GeoPref Test consisted of 28 different dynamic geometric and dynamic social scenes which changed simultaneously across the 62.22 second movie. All social and non-social stimuli are presented in eFigure 2. Total video and scene length are provided in the figure legend.

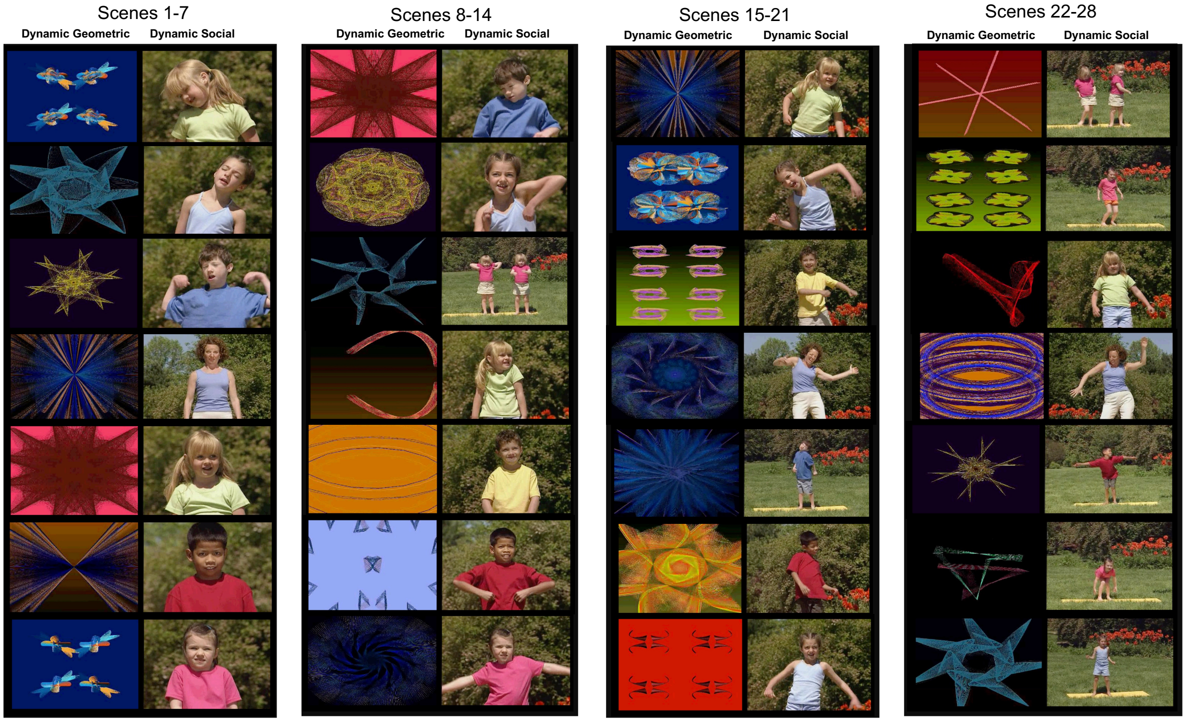

eFigure 2. Dynamic geometric and social stimuli which make up the 'GeoPref Test'. The 'GeoPref Test' consisted of a total 28 different dynamic geometric (left) and dynamic social scenes (right). Social images courtesy of Gaiam Americas Inc., Copyright 2003, Gaiam Americas, Inc. To control for any biases that might occur due to spatial location of the social and geometric images, the side of stimulus presentation (left/right) varied across subjects. Each scene was 0.7-3.8 seconds in duration. Average scene duration was 2.23 seconds. Geometric and social images played and changed simultaneously.

Supplemental Results

Visual Attention Patterns Among an Independent Sample of 1,419 Toddlers

Statistical analyses were also conducted for the new, independent sample of 1,419 toddlers. Results indicated significant differences in the amount of time a toddler fixated on DGI based on diagnostic group membership ($F(6,1412)=43.74$, $p<0.0001$). Toddlers with ASD exhibited the highest percent fixation to DGI compared to all other toddler types (ASD 95% CI [40.02, 44.00] vs. ASD-Feat 95% CI [26.38, 35.94], mean difference: 10.86 ± 2.77, $p<0.001$, $d=0.45$ 95% CI [0.22, 0.68]; ASD vs. GDD 95% CI [24.74, 32.95], mean difference: 13.17 ± 2.89, $p<0.0001$, $d=0.55$ 95% CI [0.34, 0.76]; ASD vs. LD 95% CI [19.84, 25.17], mean difference: 19.51 ± 6.92, $p<0.0001$, $d=0.85$ 95% CI [0.67, 1.02]; ASD vs. Other 95% CI [19.61, 26.07], mean difference: 19.17 ± 6.81, $p<0.0001$, $d=0.82$, 95% CI [0.61, 1.02]; ASD vs. TD 95% CI [20.54, 24.27], mean difference: 19.60 ± 7.34, $p<0.0001$, $d=0.89$ 95% CI [0.75, 1.03]; ASD vs. TypSibASD 95% CI [18.45, 29.50], mean difference: 18.03 ± 8.12, $p<0.0001$, $d=0.75$ 95% CI [0.41, 1.09]). Preference for DGI was also high for the ASD-Feat group (ASD-Feat vs. TD mean difference: 8.74 ± 4.57, $p<0.05$, $d=0.48$ 95% CI [0.24, 0.73]; ASD-Feat vs. LD mean difference: 8.65 ± 4.15, $p<0.05$, $d=0.46$ 95% CI [0.19, 0.73]). TD, LD, GDD, TypSibASD, and toddlers categorized as Other exhibited a stronger preference for DSI, and preference strength was comparable between groups. Scatterplots showing group differences, pairwise comparison of effect sizes, and validation statistics as a function of age are provided in eFigure 3.

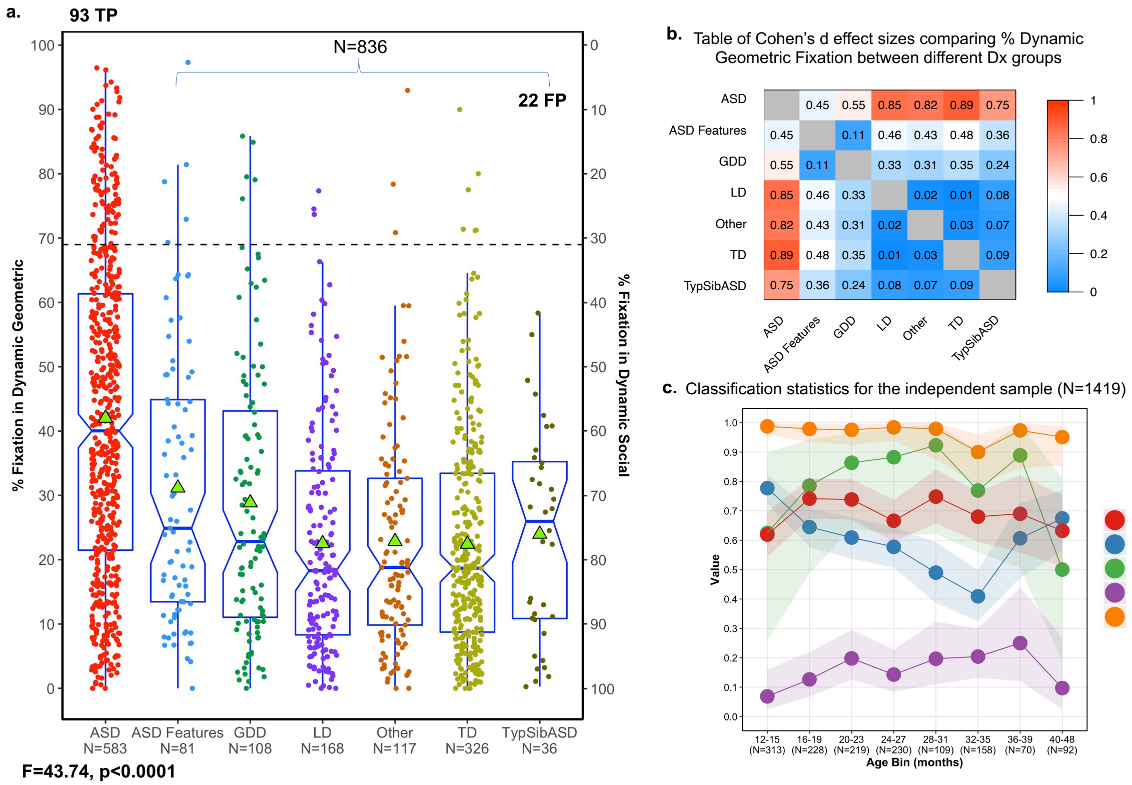

eFigure 3. GeoPref Test performance in an independent sample of toddlers across a range of diagnostic groups (N=1,419). (a) Boxplots illustrating percent fixation to dynamic geometric (DGI) or dynamic social images (DSI) for 1,419 toddlers independent of our previous work. Boxplots show median and first/third quartiles. Notch ranges indicate 95% confidence intervals for the median. Green triangles indicate mean percent fixation. F statistic based on a one-way ANOVA comparing percent DGI fixation across diagnostic groups. **(b)** Matrix of Cohen's d effect sizes obtained from pairwise comparisons of percent fixation to DGI. **(c)** Validation statistics for classification of toddlers as ASD vs. non-ASD using a 69% fixation threshold, stratified across 8 age bins (in months). ASD: Autism Spectrum Disorders, ASD-Feat: ASD Features, GDD: Global Developmental Delay, LD: Language Delay, TD: Typically Developing, TypSibASD: Typical Sibling of subject with ASD, AUC: Area under the curve, Neg. Pred. Val.: Negative Predictive Value, Pos. Pred. Val.: Positive Predictive Value.

Visual Attention Patterns Stratified by Ethnicity, Race, and Sex

GeoPref Test performance was stratified by sex, ethnicity, and race. Across diagnostic categories, percent DGI fixation did not differ between males and females (eFigure 6). Among ASD toddlers, differences in percent fixation to dynamic geometric images were observed between Hispanic/Latino and non-Hispanic/Latino toddlers (eFigure 4, One-Way ANOVA for ASD toddlers: $F(2,722)=4.09$, $p=0.017$, $d=0.23$), and between Caucasian and Other/Unknown/Not Reported toddlers (eFigure 5, $F(6,718)=3.94$, $p<0.001$, $d=-0.39$).

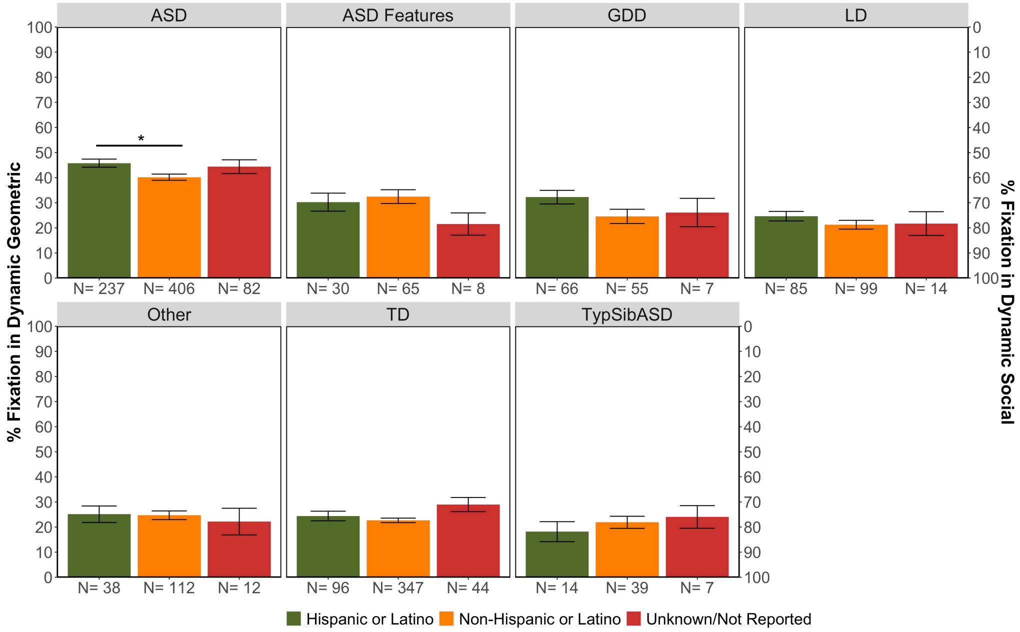

eFigure 4. GeoPref Test performance stratified by ethnicity. Bar graphs illustrating average percent fixation to dynamic geometric or dynamic social images for 1,863 toddlers independent of our previous work. Error bars indicate standard error of the mean. ASD: Autism Spectrum Disorders, ASD-Feat: ASD Features, GDD: Global Developmental Delay, LD: Language Delay, TD: Typically Developing, TypSibASD: Typical Sibling of subject with ASD, N: number of subjects, * p<0.05.

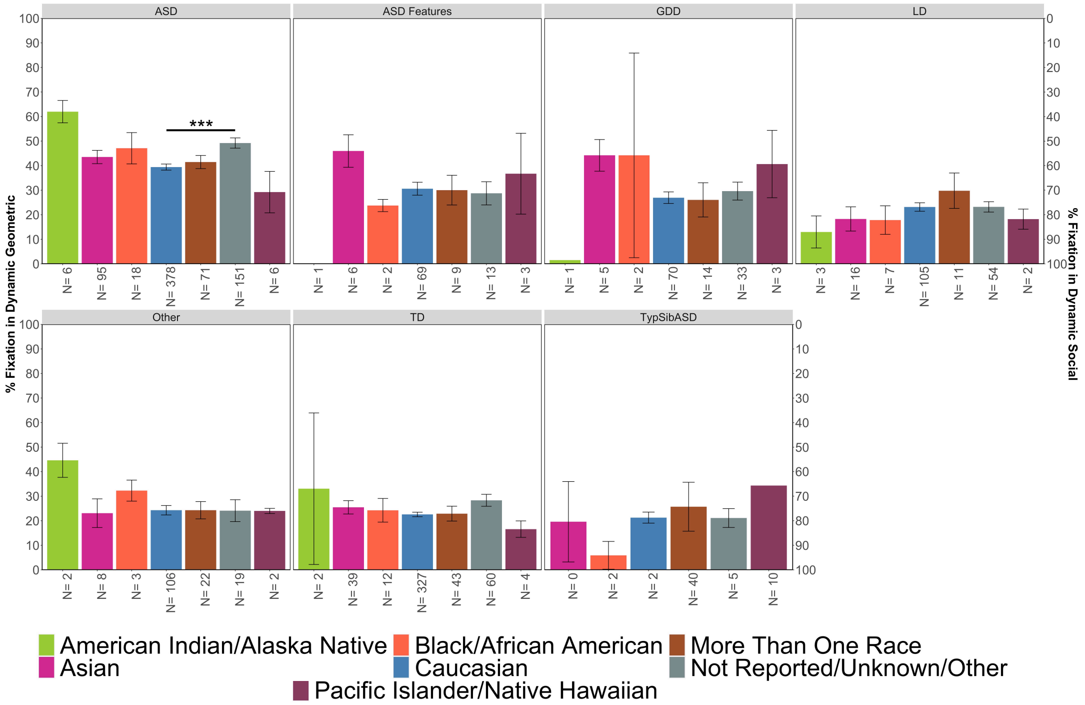

eFigure 5. GeoPref Test performance stratified by race. Bar graphs illustrating average percent fixation to dynamic geometric or dynamic social images for 1,863 toddlers independent of our previous work. Error bars indicate standard error of the mean. ASD: Autism Spectrum Disorders, ASD-Feat: ASD Features, GDD: Global Developmental Delay, LD: Language Delay, TD: Typically Developing, TypSibASD: Typical Sibling of subject with ASD, N: number of subjects, ***p<0.001.

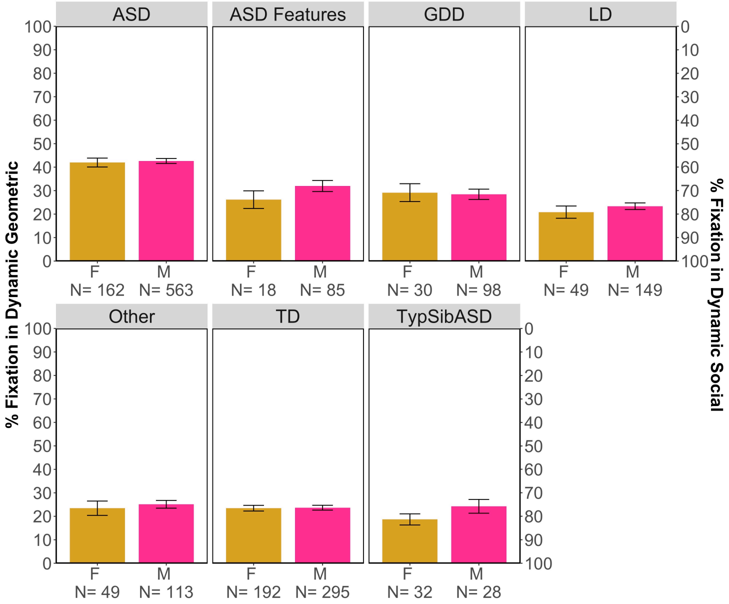

eFigure 6. GeoPref Test performance stratified by sex. Bar graphs illustrating percent fixation to dynamic geometric or dynamic social images for 1,863 toddlers independent of our previous work. Error bars indicate standard error of the mean. ASD: Autism Spectrum Disorders, ASD-Feat: ASD Features, GDD: Global Developmental Delay, LD: Language Delay, TD: Typically Developing, TypSibASD: Typical Sibling of subject with ASD, F: Female, M: Male, N: number of subjects.

Relationships Between DGI Fixation Levels and Clinical Measures Among the Full Set of 1,863 Toddlers

All clinical measures were significantly correlated with percent DGI fixation within ASD toddlers. Some significant relationships were observed for toddlers with other delays such as global developmental delay, but were not as consistent as was found within the ASD group. Interestingly, there was a lack of significant correlations between percent DGI fixation and most clinical measures among TD and TypSibASD toddlers. See eTable1.

**Correlations Coefficients Between Clinical Measures and Percent DGI Fixation**							
	ASD	ASD-Feat	GDD	LD	Other	TD	TypSib ASD
**ADOS**							
SA/CoSo	0.31****	-0.02 n.s.	-0.02 n.s.	0.11 n.s.	0.13 n.s.	0.02 n.s.	-0.05 n.s.
RRB	0.21****	0.25*	0.08 n.s.	0.11 n.s.	0.24**	-0.07 n.s.	0.15 n.s.
Total Score	0.33****	0.06 n.s.	0.01 n.s.	0.14*	0.19*	-0.002 n.s.	0.03 n.s.
**Mullen**							
Expressive Language	-0.26****	-0.15 n.s.	-0.17 n.s.	-0.12 n.s.	-0.23**	-0.04 n.s.	-0.12 n.s.
Receptive Language	-0.27****	-0.08 n.s.	-0.17 n.s.	-0.04 n.s.	-0.17*	-0.02 n.s.	-0.04 n.s.
Fine Motor	-0.19****	-0.28****	-0.25**	-0.15*	-0.24**	-0.004 n.s.	-0.12 n.s.
Visual Reception	-0.26****	-0.29*	-0.13 n.s.	-0.13 n.s.	-0.27***	0.13**	0.07 n.s.
Early Learning Composite	-0.29****	-0.21*	-0.21*	-0.15*	-0.25**	0.02 n.s.	-0.09 n.s.
**Vineland**							
Socialization	-0.29****	-0.14 n.s.	-0.18*	-0.16*	-0.14 n.s.	-0.07 n.s.	0.03 n.s.
Communication	-0.27****	-0.13 n.s.	-0.15 n.s.	-0.11 n.s.	-0.17*	0.01 n.s.	-0.02 n.s.
Motor	-0.22****	-0.15 n.s.	-0.17 n.s.	-0.01 n.s.	-0.20*	-0.04 n.s.	-0.04 n.s.
Daily Living	-0.24****	-0.14 n.s.	-0.09 n.s.	-0.13 n.s.	-0.13 n.s.	-0.04 n.s.	0.11 n.s.
Adaptive Behavior Composite	-0.29****	-0.17 n.s.	-0.19*	-0.09 n.s.	-0.17*	-0.04 n.s.	0.06 n.s.

eTable 1. DGI fixation levels are strongly correlated with clinical measures among ASD toddlers (N=1,863). Table of Pearson's r values representing the correlation between % fixation DGI and all clinical measures and associated subscales. ASD: Autism Spectrum Disorders, ASD-Feat: ASD Features, GDD: Global Developmental Delay, LD: Language Delay, TD: Typically Developing, TypSibASD: Typical Sibling of subject with ASD; ADOS: Autism Diagnostic Observation Scale, SA/CoSo Score: Social Affect/Communication Social Score, RRB Score: Restricted and Repetitive Behavior Score. *p<0.05, **p<0.01, ***p<0.001, ****p<0.0001, n.s.: not significant.

Validation Statistics as a Function of DGI Fixation Threshold

In the present study, a 69% fixation to dynamic geometric images was selected as a cutoff for test validation as this provides optimal specificity. Clinicians may use the following table (eTable 2) to tune the GeoPref Test to their desired level of specificity.

Fixation Threshold % Geometric	Sensitivity	Specificity
0%	99.53%	1.52%
1%	99.22%	3.03%
2%	98.64%	5.10%
3%	97.56%	7.69%
4%	96.20%	10.06%
5%	95.28%	12.44%
6%	94.55%	15.01%
7%	93.28%	17.54%
8%	91.88%	20.26%
9%	90.22%	22.87%
10%	88.79%	25.07%
11%	88.14%	28.04%
12%	87.18%	31.09%
13%	85.24%	33.44%
14%	84.18%	36.58%
15%	82.57%	38.62%
16%	80.41%	40.77%
17%	79.02%	43.48%
18%	78.08%	46.59%
19%	77.41%	49.33%
20%	76.60%	51.22%
21%	75.38%	52.93%
22%	74.24%	54.89%
23%	72.97%	56.61%
24%	71.70%	58.29%
25%	70.17%	59.79%
26%	68.73%	61.42%
27%	67.43%	63.23%
28%	66.54%	65.17%
29%	65.45%	66.28%
30%	63.96%	67.40%
31%	62.39%	68.53%
32%	61.06%	69.64%
33%	59.41%	71.09%
34%	58.11%	72.96%
35%	56.90%	74.64%
36%	54.83%	76.20%
37%	53.05%	77.57%
38%	51.62%	78.66%
39%	51.09%	79.87%
40%	49.94%	81.00%
41%	48.91%	82.06%
42%	47.69%	82.88%
43%	46.51%	83.67%
44%	45.36%	84.77%
45%	44.20%	85.87%
46%	43.27%	86.53%
47%	41.91%	87.18%
48%	40.45%	88.08%
49%	39.10%	88.88%
50%	37.85%	89.65%
51%	36.27%	90.56%
52%	34.81%	90.94%
53%	33.98%	91.37%
54%	33.31%	92.02%
55%	32.14%	92.56%
56%	30.90%	92.93%
57%	29.95%	93.34%
58%	28.90%	93.98%
59%	27.82%	94.32%
60%	27.10%	94.93%
61%	25.97%	95.27%
62%	24.64%	95.44%
63%	23.62%	95.86%
64%	22.68%	96.19%
65%	21.65%	96.54%
66%	20.73%	96.88%
67%	19.47%	97.12%
68%	18.05%	97.39%
69%	17.08%	97.55%
70%	16.58%	97.62%
71%	15.80%	97.86%
72%	14.96%	98.04%
73%	14.17%	98.23%
74%	13.24%	98.45%
75%	12.07%	98.50%
76%	11.13%	98.65%
77%	10.24%	98.88%
78%	9.20%	98.97%
79%	8.37%	99.22%
80%	7.73%	99.40%
81%	7.25%	99.47%
82%	6.66%	99.60%
83%	5.90%	99.60%
84%	5.23%	99.60%
85%	4.71%	99.61%
86%	4.13%	99.70%
87%	3.73%	99.70%
88%	3.23%	99.70%
89%	2.70%	99.70%
90%	2.28%	99.80%
91%	1.80%	99.80%
92%	1.37%	99.80%
93%	1.15%	99.90%
94%	1.00%	99.90%
95%	0.75%	99.90%
96%	0.43%	99.90%
97%	0.10%	99.95%
98%	0%	100.00%

eTable 2. Coordinates of the ROC Curve. Percent fixation values within dynamic geometric area of interest and associated sensitivity and specificity values based on data from 1,863 toddlers.

Validation of GeoPref Test Performance Stratified by Ethnicity, Race, and Sex

Given the large sample of toddlers examined, validation statistics were compared across ethnic categories, race, and sex (eTable 3).

Classification Statistics by Ethnicity

Ethnicity	No. Subjects	Sensitivity	Specificity	PPV	NPV	LCI	AUC	UCI
Hispanic or Latino	566	21.50%	97.00%	83.60%	63.20%	0.68	0.73	0.77
Non-Hispanic or Latino	1123	14.50%	97.50%	76.60%	66.80%	0.66	0.70	0.73
Unknown/Not Reported	174	18.30%	98.90%	93.80%	57.60%	0.65	0.72	0.80

Classification Statistics by Race

Race	No. Subjects	Sensitivity	Specificity	PPV	NPV	LCI	AUC	UCI
American Indian/Alaska Native	15	33.30%	100.00%	100.00%	69.20%	0.75	0.91	1.00
Asian	171	21.10%	97.40%	90.90%	49.70%	0.61	0.69	0.77
Black/African American	46	27.80%	96.40%	83.30%	67.50%	0.63	0.77	0.91
Caucasian	1095	13.50%	97.40%	72.90%	68.10%	0.65	0.69	0.72
More Than One Race	175	11.30%	96.20%	66.70%	61.30%	0.64	0.71	0.79
Pacific Islander/Native Hawaiian	21	0.00%	100.00%	NA	71.40%	0.21	0.51	0.82
Not Reported/Unknown/Other	340	25.80%	98.40%	92.90%	62.40%	0.70	0.76	0.81

Classification Statistics by Sex

Sex	No. Subjects	Sensitivity	Specificity	PPV	NPV	LCI	AUC	UCI
Female	532	15.40%	97.30%	71.40%	72.40%	0.68	0.73	0.78
Male	1331	17.80%	97.50%	84.00%	61.80%	0.67	0.70	0.73

eTable 3. Classification statistics for subjects across all age groups stratified by ethnicity, race, and sex using the 69% fixation threshold. PPV, positive predictive value; NPV, negative predictive value, LCI, lower confidence interval; UCI, upper confidence interval; AUC, area under the curve; NA, not applicable.

Reliability of GeoPref Test Performance in Toddlers with Multiple Eye-Tracking Sessions
Five hundred and thirty-five toddlers received a second eye-tracking session in order to evaluate test-retest reliability of the GeoPref Test. eTable 4 summarizes test intervals and reliability across multiple tests.

	Test-Retest Interval (N)				
**Diagnostic Group**	**0 – 1 mo. Immediate**	**2 – 6 mo. Short Term**	**7 – 12 mo. Intermediate Term**	**13 – 24 mo. Long Term**	**>25 mo. Very Long Term**
**ASD**	10	41	92	47	4
**ASD-Feat**	3	7	16	15	4
**LD**	2	6	15	9	1
**GDD**	0	3	5	7	1
**Other**	5	9	18	12	5
**TD**	18	27	58	65	7
**TypSibASD**	1	7	7	8	0
**Total**	39	100	211	163	22
**Absolute change score, % geo between T1 & T2**	11.80 ± 10.80	16.48 ± 13.56	19.14 ± 15.06	19.38 ± 15.01	27.98 ± 21.51
**Intraclass correlation between T1 & T2**	0.76	0.53	0.42	0.42	0.08
**Intraclass correlation p-value**	p<0.0001****	p<0.0001****	p<0.0001****	p<0.0001****	p=0.26$^{n.s}$
**Paired t-test between T1 & T2: T-statistic**	-0.72	-1.92	-2.74	-3.15	-3.92
**Paired t-test p-value**	0.474$^{n.s}$	0.057$^{n.s}$	0.0064**	0.0018**	p<0.0001****

eTable 4. GeoPref Test performance is highly reliable up to 24-months following the first eye-tracking session. Table details number of subjects in each diagnostic category who have test-retest data at varying intervals. ASD: Autism Spectrum Disorders, ASD-Feat: ASD Features, GDD: Global Developmental Delay, LD: Language Delay, TD: Typically Developing, TypSibASD: Typical Sibling of subject with ASD, n.s.: not significant, ** p<0.01, ****p<0.0001.
